# Navigation outside of the box: what the lab can learn from the field and what the field can learn from the lab

**DOI:** 10.1186/2051-3933-2-3

**Published:** 2014-02-03

**Authors:** Lucia F Jacobs, Randolf Menzel

**Affiliations:** Department of Psychology, University of California, Mailcode 1650, Berkeley, CA 94520-1650 USA; Institut für Biologie, Freie Universität, Königin-Luise-Strasse 28/30, 14195 Berlin, Germany

**Keywords:** Cognitive map, Landmark, Geometry, Locomotion, Hippocampus, Parallel map theory

## Abstract

Space is continuous. But the communities of researchers that study the cognitive map in non-humans are strangely divided, with debate over its existence found among behaviorists but not neuroscientists. To reconcile this and other debates within the field of navigation, we return to the concept of the parallel map theory, derived from data on hippocampal function in laboratory rodents. Here the cognitive map is redefined as the integrated map, which is a construction of dual mechanisms, one based on directional cues (bearing map) and the other on positional cues (sketch map). We propose that the dual navigational mechanisms of pigeons, the navigational map and the familiar area map, could be homologous to these mammalian parallel maps; this has implications for both research paradigms. Moreover, this has implications for the lab. To create a bearing map (and hence integrated map) from extended cues requires self-movement over a large enough space to sample and model these cues at a high resolution. Thus a navigator must be able to move freely to map extended cues; only then should the weighted hierarchy of available navigation mechanisms shift in favor of the integrated map. Because of the paucity of extended cues in the lab, the flexible solutions allowed by the integrated map should be rare, despite abundant neurophysiological evidence for the existence of the machinery needed to encode and map extended cues through voluntary movement. Not only do animals need to map extended cues but they must also have sufficient information processing capacity. This may require a specific ontogeny, in which the navigator’s nervous system is exposed to naturally complex spatial contingencies, a circumstance that occurs rarely, if ever, in the lab. For example, free-ranging, flying animals must process more extended cues than walking animals and for this reason alone, the integrated map strategy may be found more reliably in some species. By taking concepts from ethology and the parallel map theory, we propose a path to directly integrating the three great experimental paradigms of navigation: the honeybee, the homing pigeon and the laboratory rodent, towards the goal of a robust, unified theory of animal navigation.

## Introduction

How humans and other animals model their external world for spatial navigation has captured the imagination of scientists from ethology, ecology, cognitive and comparative psychology, neuroscience, robotics and artificial intelligence. Goal-directed movement across space thus has the potential to integrate these disciplines conceptually. The concept of the cognitive map is one common to all, yet we lack a synthesis or agreement as to its precise nature and characteristics. The goal of our review is to propose such a synthesis, to increase the power and scope of interdisciplinary communication related to the issue of the cognitive map.

The cognitive map, in its modern definition, represents the most flexible use of spatial information to solve a simple problem: the animal must devise a novel solution to orient to its goal [1]. The definition of this problem in the laboratory is that an animal needs to orient between its location and its goal using a novel route that has been simulated from its prior knowledge of the space. For example, a rat initially trained to reach the goal from a certain start location in a well-learned maze could be tested from a novel start location. In the field, this problem is defined similarly: a displaced animal needs to orient from a novel release point to its experienced home area. In both the lab and the field, the animal must be able to recognize a cue at the release site that marks its spatial relation to the goal.

Using demonstrations of flexible detouring and shortcutting as evidence for a map-like organization of remembered travels became the focus of research in many disciplines, from molecular neuroscience to animal behavior, movement ecology and human cognition. The scientists who first formulated the concept of map-like behaviors in animals did so with full awareness that they were proposing a new and possibly more complex mechanism for spatial navigation. In what turned out to be a remarkable convergence of insight, a comparative psychologist using lab rats and an ethologist studying homing pigeons in the field simultaneously articulated the idea that animals were orienting using map-like information. In Berkeley, Edward Tolman summarized decades of work on the purposive behavior of the laboratory rat with his manifesto on the ability of this animal to form a representation of its external environment [2]. In Wilhelmshaven, Gustav Kramer (the first to demonstrate the use of a sun compass in captive starlings) concluded from his work on the navigation of homing pigeons that the feats they accomplish could only be explained by their possession of topographic knowledge organized as a model of the world, integrated with knowledge of compass information [3]. Donald Griffin then encapsulated Tolman’s concept of the cognitive map and Kramer’s insights on pigeon navigation with his proposed classification of bird navigation modes, with Type III being true navigation: the ability to orient towards a goal, regardless of its direction, based on mechanisms other than recognition of landmarks [4].

In the 1970’s, John O’Keefe and Lynn Nadel resurrected Tolman’s concept of the cognitive map [2] to interpret O’Keefe’s discovery of place cells in the rodent hippocampus, the first evidence that hippocampal neurons are activated by specific locations in space. This culminated in their 1978 synthetic theory and book, “The Hippocampus as a Cognitive Map” [5]. Thus for approximately thirty years neuroscientists have incorporated the concept of the cognitive map into theories of spatial orientation. The use of this theoretical framework continues to catalyze research in this field, and led to the discovery of other specialized spatial function cells in the hippocampal formation, such as the subicular boundary cells [6], subicular head direction cells and, most spectacularly, the medial entorhinal grid cells [7]. Recently, hippocampal “time cells” were described that code more specifically for time or distance moved, indicating that the hippocampal network represents both the timing and distances of experienced locations [8]. Furthermore, planning of future shortcuts between learned locations can be read from the phase relations of hippocampal place cell activity, demonstrating the pre-play of future actions and their expected neural correlates [9]. All of these components should theoretically play different roles in the construction of a map-like representation, whose properties include retaining a memory of spatial relationships even in the absence of sensory input, as in ‘replaying’ during REM sleep [10]. Methods continue to be developed at a fast pace, including virtual reality spatial arenas, simultaneous recording from more than 150 neurons and even genetic manipulations that can selectively stimulate or silence specific neurons via optophysiological means in actively navigating rodents. Such studies continue to demonstrate that elements of map-like behavior can be traced in the laboratory rodent to subtle properties of the underlying neural circuitry [11]. Finally, humans are becoming a valuable study species, with the development of ever more sophisticated virtual reality spatial tasks combined with functional neuroimaging methods. Here, too, the concept of the cognitive map and navigational principles derived from studies of rodents are being used to understand brain function in humans [12].

### The question of non-mapping solutions

**Figure 1:**
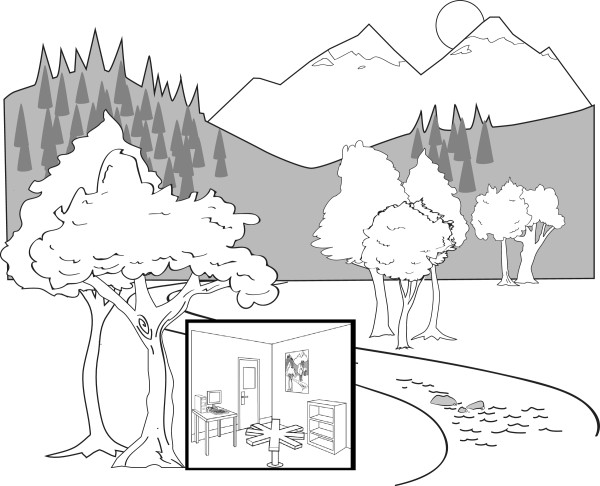
**A schematic comparison of the spatial scale and extended cues of a representative navigation environment compared to the cues available to the animal navigating a radial-arm maze in a representative laboratory test room.**

Yet the question of the cognitive map, even in the lab rat, remains much more controversial among behaviorists than among neuroscientists. One reason for such skepticism is the question of complexity and parsimony. The cognitive map solution is one type of place strategy to return to a learned location, but there are also other methods [1, 13, 14]. Without knowing the neural and computational bases of non-map mechanisms we cannot say that they are simpler, but they are certainly different. In the present review, we will propose that an important reason for this controversy is the difference in spatial scale between the field and the lab (Figure 1).

Nonetheless, in both contexts the cognitive map demands that an animal can accurately correct its goal trajectory after experimental displacement. The cognitive map is thus an example of a type of place strategy in which a goal location is defined only by remote sensory cues. But it must make this correction without relying solely on other strategies or mechanisms. For example, an animal could return to a known location using only dead reckoning, which supports a low-risk and rapid return to the place of origin by path integration. It could use a cue strategy, where the goal is defined by a unique cue that has a fixed location in relation to the goal, either an object or the structure of the panorama surrounding the goal. Another stand-alone strategy is a response strategy, in which a navigator recalls and executes memorized body movements that will bring it to its goal [15].

Familiar landmarks or beacons that can be perceived at both the release point and the goal location allow the animal to navigate by creating and independently storing multiple vectors derived from path integration that lead to the goal [16]. Panorama matching also uses a memorized array of landmarks (i.e., image matching), with the difference that cues are learned as a single unit rather than as independent landmarks, a more flexible strategy [17]. Because panorama matching relies on a memorized view, however, it is similar to orientation to landmarks. It has been studied intensively in ants and bees close to their nest sites, where it can explain much of the orientation seen in the field in many ant species [18, 19]; using a collection of vectors, an animal can return to its goal [20]. Path integration could reflect a mechanism of egocentric navigation by which distance-weighted directional components are continuously integrated and a running estimate (minus 180°) provides the vector to the starting point, irrespective of landmarks. It could also be understood as reflecting neural operations that compute the shortest trajectories between the current location and the goal in reference to a geometric layout of spatial codes, such as place cells and grid cells in the hippocampus that are defined by landmarks [21].

Thus vector addition can either be a rather simple form of computing two or more egocentric trajectories that are stored in working memory, or it can indicate processes on the level of the neural representation of a cognitive map. The relationship between landmark-based and vector-based navigation can be tested using clock-shift experiments. For example, recent studies of clock-shifted pigeons released at a familiar site have reported that individual birds vary in their use of familiar landmarks, using them as direct cues of location or using them as cues that have been associated with a compass direction [22]. Even pigeons released in view of the loft will deviate from a direct flight towards the loft, according to the time-shifted sun compass [23]. It is noteworthy that a pigeon’s ability to direct its flight toward its home loft, even when released from an unfamiliar site, is based on being able to localize the direction of home in relation to its current location by using a sun or a magnetic compass [24]. However, in nature, navigation is often connected to exploratory behavior [25, 26] and the animal’s performance improves with knowledge about the environment, information which may be encoded in a cognitive map. By contrast, pigeons can orient in the correct direction from unfamiliar locations but do not appear to have an expectation of the correct distance [27]. Similar evidence for knowing direction but not distance has been reported for wild red squirrels homing from an unfamiliar site, as squirrels appear to travel a certain distance in the correct direction and then return to the unfamiliar release point [28].

In summary, animals use a variety of redundant mechanisms to ensure their success in navigation, a behavior critical for survival. Animals rely heavily on non-mapping solutions yet there is also evidence for more flexible, mapping solutions [1]. One of the goals of our review is to discuss why mapping solutions are rarely observed. The first reason may well be the lack of tools to measure navigation across natural scales of movement.

### New tools, new insights

As with other revolutionary tools that have increased the scope of human perception, such as the microscope, telescope or recording electrode, the science of animal movement and the debate about the cognitive map are continually influenced by the development of new technology. After a period of few advances since the introduction of radiotelemetry for large-bodied animals in the 1960’s, recent years have seen great advances in methods. Methods such as harmonic radar and Global Positioning System (GPS) data loggers have revolutionized the study of animal navigation [29]. One obstacle to a grand synthesis of navigational strategies is that each species studied contributes important pieces of the larger picture but nonetheless has navigational strategies adapted to its particular life history constraints. It is therefore crucial to study navigation across a wide range of species, yet this has only recently been possible with animals as small as insects.

Central-place foragers such as the honeybee must remember not only their nest location but also the location of multiple food patches. When leaving either the hive or a food patch, honeybees perform a characteristic scanning behavior and from this behavior they learn the panorama of landmarks. They use these images to orient to locations on this scale. The question of whether hymenopteran insects (bees and ants) use this same mechanism, the recollection of the image of landmarks and panoramas, for all scales of localization, including the largest scale of distant panoramas [17], is not clear, as the evidence is only indirect. For example, ants traveling in a visually cluttered environment appear to use only the image-matching strategy [18]. However, ants in these experiments and honeybees in earlier experiments [17] had established a fixed route by multiple trials (either walking or flying, respectively). Thus their performance under these conditions may rely on a different mechanism of spatial memory than that used after initial exploratory behavior, when the dead reckoning vectors associated with landmarks were being established. It would be interesting and important to determine whether ants, like honeybees, behave differently when examined after only exploratory runs; without these data, it is still unclear whether panorama-matching is the only mechanism available to them for navigation on a large scale.

#### Map-like behavior in insects

**Figure 2:**
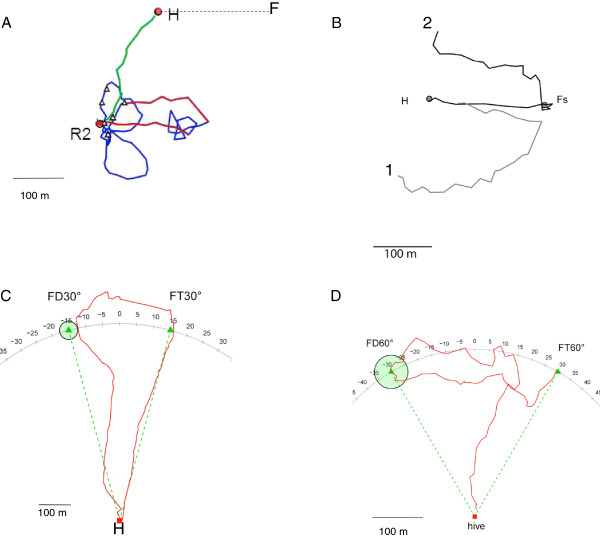
**Radar tracks of navigating honeybees under four different conditions. A**. Dance-directed flights and homing flights. The test bee followed a dancing bee that indicated a feeding place 200 m east of the hive. When leaving the hive she was released at the site R. She flew 200 m east (lower red line), searched briefly at the terminal of her dance-directed flight, and returned to the release site (upper red line) where she searched in systematic loops and then flew straight back to the hive (green line) [32]. **B**. Homing flights via the feeder. In this experiment bees foraged at the feeder (F) 200 m east of the hive. Two test bees were captured when preparing to fly back to the hive (H) and released at either 300 m south or north of the hive. After search flights they flew first to the feeder and then back to the hive [32]. **C**. Short-cutting flights between experienced and communicated locations. The bee was trained to a feeder (FT, triangle) 650 m north of the hive (H). After one day of no food at this feeder, she attended a dance of a bee that indicated the place FD (triangle with green circle), 650 m away from the hive and under 30° to the hive-feeder direction. She flew first to FT and then crossed over to FD, from there returning to the hive [33]. **D**. Short-cutting flights depend on the absolute distance between experienced and dance-indicated place. This is the same experimental design as in C, above, but with only 300 m distance between hive and FT or FD. Here bees performed short-cutting flights between FT and FD in the 60° tests as well, not only in the 30° test with distances of 650 m between hive and FT or hive and FD [33].

Harmonic radar has proved critical to resolving the question of map-like behavior and spatial cognition in flying insects, which, in the absence of this technology, was controversial [30, 31]. It is the only method by which the paths of individual insects, such as the honeybee, can be tracked with accuracy across the scale of natural landscapes. In 2005, Menzel and colleagues demonstrated that the honeybee, tracked over natural distances in the field, indeed shows map-like behavior [32]. Figure 2 illustrates results from bees in a second series of experiments [33]. In these experiments, the data from harmonic radar demonstrated that a honeybee recruited to a food source maintains multiple memories of locations. Moreover, these memories are based on past experiences and the newly acquired information from another bee’s waggle dance. A bee’s use of this information is flexible, changing with the distance and angle between feeders. The bee is also able to incorporate both her own experience and newly acquired public information. Because both of these sources of information are known, the bee’s flights can be categorized as goal-directed toward an individually experienced and learned location and a dance-communicated location. Under these circumstances, bees have been observed to flexibly shortcut between the locations of multiple feeders and the hive. Moreover, their propensity for shortcutting varies with the economic costs; that is, they are more likely to shortcut when two feeders are close together than when they are far apart. In this series of studies, the field site was also chosen to be free of large landmarks that could have been used as beacons; panorama matching to calculate these shortcuts was also excluded. Thus these new studies of flying honeybees, tracked using harmonic radar, have reframed the problem of the cognitive map in insects in a completely new light, such that map-like behavior is now a strongly supported hypothesis for navigation in honeybees.

An alternative view has been presented by Cruse and Wehner [20], who argue that vector addition of three vectors (outbound and inbound vector of the learned flight and outbound vector of dance-communicated information), together with a change in motivation (outbound or inbound), is sufficient to explain the shortcutting behavior of bees. However, such a proposal assumes that the memory of the experienced space lacks information about landmarks, a rather unlikely assumption, given the well-documented guidance of bees by landmarks. It should be possible to decide between these two interpretations based on behavioral data by disconnecting compass-based vector addition from homing. In both birds and honeybees this is possible by clock-shifting the animals, as vectors are related to the sun compass. Pigeons appear to rely on a compromise between navigation related to the sun compass and to the magnetic compass [24]. In honeybees, the vector flight, the initial flight path (Figure 2), depends on the sun compass only in a relatively featureless environment, whereas this flight does not depend on the sun compass when the bee is performing homing flights and vector flights in a rich environment with extended landmarks. Similarly, in a more familiar area, pigeons also re-orient more to their memory of a familiar topography than to information from a magnetic compass [34]. At the neural level a decision between the two interpretations requires knowledge about the coding of space, information that is not yet available for birds and insects.

In conclusion, there are several simple but powerful non-mapping mechanisms that an animal can use to home from a novel release point. Yet some results cannot be explained by these mechanisms, as the honeybee can flexibly use and recombine these vectors. To do this, bees must be able to embed the vectors into a relational geometric representation or perform vector integration on multiple vectors in working memory [32, 33]. Thus the harmonic radar method, supplying for the first time data from flying insects foraging over natural scales of movement, offers clear evidence for a map-like strategy in a non-vertebrate.

#### Navigation in flying vertebrates

What harmonic radar did for our insight into the cognition of flying insects, radiotelemetry and Global Positioning Systems (GPS) have done for our insight into the navigational mechanisms of flying vertebrates, in particular domesticated homing pigeons [34–37]. The development of miniaturized GPS logging devices has greatly increased the quantity and quality of movement data for pigeons, particularly enriching our knowledge of the behavioral mechanisms underlying navigation in the homing pigeon [26, 35, 38–40]. Because this technology allows tracking in three dimensions, it will also be possible to estimate the angular view of landmarks during the animal’s navigation, such that future studies may be able to recreate the visual experience of navigating birds while they are making navigational decisions.

Tracking methods are also being used in sophisticated ways to study spatial strategies in wild species during natural migratory behavior. Wikelski, Thorup and colleagues have radiotracked migrating birds using small aircraft for large-scale field experiments. For example, in a study of white-crowned sparrows, experienced adults were caught on their southward journey from Alaska in the state of Washington and displaced 3700 km to the state of New Jersey [41]. Experienced adults were able to correct their route, but first-time migrating juvenile sparrows, given the same displacement, could not correct their trajectory to their winter habitat. The ability of adults, but not juveniles, to steer toward a remembered location thousands of kilometers from their location of displacement must therefore be due to experience and/or brain development. The difference allows the older animals to use a navigational map to extrapolate the direction of the goal from this position.

GPS has also made possible the first fine-grained analysis of homing after displacement in a flying mammal, the Egyptian fruit bat [42]. Bats released in a novel location were able to orient to their home roost, providing the first evidence of map-like behavior in a mammal in the field. Thus new technology again has allowed researchers to collect the critical data to detect map-like behavior in a previously unstudied taxon.

## Review

### What the field can learn from the lab

These advances are ushering in a golden age for the study of animal movement, as navigation in the field becomes amenable to measurement that has the precision and accuracy formerly possible only in the lab. Yet this abundance of data, similar to that seen in other fields with new tools, must be harnessed using conceptual advances, as the volume of data increases rapidly with the application of GPS technology and new, even more data-rich methods under development [29]. Because the field of the neuroscience of navigation in rodents is well advanced, here we consider what concepts from the lab could be usefully brought to bear on questions of animal movement in the field.

The majority of our knowledge on animal navigation comes from three paradigms: the homing pigeon, the honeybee and the laboratory rodent (rat, mouse or hamster) [15]. Two of these three are flying species, navigating across natural landscapes, an issue that will become important later. But we have the best data on the neural mechanisms of navigation in the rodents. What can the rodent data tell us about pigeons and honeybees?

We can start with the neural homology between the more closely related vertebrate species, the lab rat or mouse (both rodents in the Family Muridae) and the homing pigeon, of which there is also a lab strain. The same brain structure, the hippocampus, mediates spatial orientation in all species [43]. The size of the hippocampus and its homologues is generally predictive of the degree to which a vertebrate species needs to construct maps of space that are flexible and can be used to derive shortcuts, often in response to social competition such as competition for mates or stored food caches [44].

This similarity in hippocampal function might shed light on an important unanswered question in pigeon navigation: the degree to which the two different navigational mechanisms of the pigeon are independent or integrated. The two mechanisms, the navigational map and the familiar area map, are well known and well described; for clarity in the present discussion and to simplify the issues for the general reader, we follow Holland (following the summary in [16]), who summarizes the seminal work of pioneers in this field, including Papi [45], Wallraff [27], Gagliardo [46] and Bingman [43].

The navigational map (hereafter NAV) is the mechanism by which a pigeon is able to orient homeward from an unfamiliar location. In such circumstances, as long as the pigeon has access to olfactory inputs at the release site [46], it can orient homeward. The pigeon does not appear to have an estimate of distance to its goal, however, but instead uses the NAV to identify the direction that is necessary to contact landmarks within its familiar area map (hereafter FAM). Once it perceives familiar landmarks, it then orients to its goal, the loft, in relation to these landmarks [16]. Olfactory deprivation disassociates the two homing mechanisms, impairing NAV but not FAM [46]. Another disassociation between the two maps is seen in clock-shift manipulations. These produce deflections in orientation comparable to the expected deflection in birds released from unfamiliar locations, i.e., orienting to the NAV, but much smaller than those seen in birds released at familiar sites, i.e., orienting to the FAM. In the latter case, pigeons are presumably piloting to the goal within the array of known landmarks [16].

As already noted, there are clear homologies and similarities between spatial and hippocampal function in free-flying homing pigeons and rats navigating in laboratory mazes. Bingman and colleagues in particular have articulated the similarities between the effect of hippocampal lesion (hereafter HP- if lesioned, HP+ if intact, following a notation similar to that used by Wallraff [27]) on navigation in these two seemingly dissimilar research paradigms [47, 48]. Moreover, this group has now provided evidence for place-specific activity in hippocampal neurons of pigeons navigating a laboratory maze [49], similar to place cells in the rat hippocampus. The consensus that has been reached from all of these studies is that the FAM, which is impaired in HP- pigeons, is similar if not homologous to the cognitive map encoded by the rat hippocampus. In both cases, the HP- animal cannot flexibly reorient to its goal using a novel route, that is, using a novel ordering of the familiar landmarks. Thus the disorientation of a HP- pigeon within its FAM in the field appears to be directly comparable to the disorientation of a HP- rat in the water maze, an open pool of water containing a subsurface, hidden platform [50]. Like the HP- pigeon, the HP- rat can orient to the platform using an already remembered route but cannot construct a new route, as a HP+ rat can do easily [51]. The HP- pigeon similarly has an impairment using the FAM, but not the NAV.

But what is more surprising is that the HP- pigeon is not impaired in orientation using the NAV, i.e., when released in an unfamiliar site. Of course, an unfamiliar site cannot be completely unfamiliar for a pigeon to orient to the homeward direction upon release. To do this, the pigeon must be able to detect an odor that it has previously encountered, specifically, an odor whose identity has been associated with the natural movement of wind. Only when these conditions are met—that the pigeon has access to odors at the release point and the pigeon has had experience associating wind direction with that odor—will the pigeon show unimpaired initial orientation at a site that is completely novel visually, if not in terms of olfaction. In addition to olfaction, there may be other sensory modalities involved in the NAV, but only olfaction is both sufficient and necessary [27].

### Parallel maps in mammals

Thus there is a general consensus that there are dual processes that must work together for the pigeon to express the full repertoire of flexible navigation, whether released in a familiar location, using the FAM, or released in an unfamiliar location, using the NAV. Subsequent to the development of this multiple map model for pigeons, developed most notably by Hans Wallraff [27], a similar development occurred in the world of mammalian behavioral neuroscience, with the development of the parallel map theory (PMT) of Jacobs and Schenk [52]. This model of navigation was also developed in response to patterns of neural double disassociations between parts of the hippocampus and spatial behaviors. In the case of the rodent hippocampus, there were many more studies to draw upon, studies in which specific areas of the hippocampus were lesioned, using an array of methods ranging from genetic to pharmacological to actual physical lesions. There had also been the development, since the first experiments of Tolman, of sensitive assays for different spatial functions, such as Olton’s radial arm maze and Morris’s water maze [15].

What emerged from this literature is that there were behavioral disassociations between lesions of hippocampal subfields, such that different subfields appeared to encode different classes of cues, termed directional cues and positional cues, where path integration was defined as an internal directional cue. External directional cues included compasses (e.g., sun compass, magnetic compass) but also polarized stimuli, such as gradients (e.g., olfactory), as well as extended stimuli (e.g., river, line of vegetation) or polarized geometrical shapes (e.g., rectangle). Distant cues, terrestrial objects and even celestial objects such as the sun or patterns of polarized light, were also defined as directional cues. Directional cues had an important property: as vectors, a navigator can extrapolate a bearing along a directional cue, either into an unknown area, or, if suddenly released into a new area, from an unknown area back to the known area. Thus, theoretically, a few directional cues could be used to create a coarse but flexible representation of space. Such directional cues were hypothesized to underlie one component, the bearing map (BE), making up one part of the cognitive or integrated map [52].

In contrast, positional cues were defined as discrete, memorized objects, whose location was close enough to the goal to be used to encode the distance of the navigator to the goal. Increasing the number of bearings, by increasing the number of positional cues, improves this accuracy in birds and mammals [53, 54]. Positional cues were hypothesized to underlie the second component of the integrated map, the sketch map (SK). It was proposed that each animal constructs a single BE that expands and increases in resolution throughout its life as more directional cues are encoded and calibrated. In contrast, SKs begin as short-term memory representations of an array of positional cues but later may be consolidated into the integrated map, where the positional cues are recoded into BE coordinates.

Based on the physiology of the rodent hippocampus, PMT further proposed that the BE and the SK were the two functions of the two components of the mammalian hippocampus, the dentate gyrus and the Ammon’s Horn, with another extra-hippocampal structure, the septum, also necessary for BE function. In addition, another subfield in the Ammon’s Horn integrates the BE and the SKs into a single integrated map, a concept similar to prior definitions of the cognitive map. After SKs are encoded by one part of the hippocampus (subfield CA1), another part (subfield CA3) integrates the BE and SKs [52, 55]. The two mechanisms, BE and SK, appear to be used independently or together, as the integrated map [52, 56].

PMT thus made several contributions to the discussion of navigation. First, it offered a hippocampal theory framed in relation to the evolution of navigation from simpler mechanisms in vertebrates. It proposed that the BE, a coarse resolution map based on directional cues, was the ancestral vertebrate navigational mechanism. The SK was evolutionary more recent and was highly developed only in mammals and birds [56]. PMT also introduced the BE concept, similar to Wallraff’s gradient map [27], to the literature on the mammalian hippocampus, which had previously been focused on maps constructed primarily from positional cues. Recent studies of synaptic plasticity in the laboratory rat are concordant with PMT. Using cues of different size and placement, plasticity in the dentate gyrus, a key BE structure, is specifically sensitive to encoding space using directional cues, while plasticity in the key SK structure (CA1) is specifically sensitive to positional cues [57]. Finally, plasticity in the integrated map structure (CA3) is sensitive to both directional and positional cues, concordant with its proposed role in PMT [58].

One reason it has not been clear to what extent PMT could be applied to non-mammalian vertebrates is that PMT is specific to hippocampal subfields and thus the homologues of the Ammon’s Horn and dentate gyrus, if they exist in other taxa, must be identified first. There is some consensus, for example, that the avian and mammalian hippocampus are homologous to the reptilian medial cortex [59, 60]. The lizard medial cortex, moreover, appears specifically homologous to the mammalian dentate gyrus [61]. The identification of hippocampal homologues in birds is more controversial [62–64]. Recent evidence suggests, however, that the ventral hippocampus of the bird, close to the septum, may be homologous to the dentate gyrus [65]. If so, then such a dentate gyrus homologue, along with the septum, might well subserve the BE function in birds as well as mammals.

### Parallel maps in birds?

Regardless of the final resolution of these structural homologies, the cognitive model of the PMT could also be useful in our efforts to synthesize concepts of navigation across taxa and paradigms. For example, the avian large scale map that is used in unfamiliar sites, the NAV, could be homologous to the mammalian BE. Likewise the avian map of familiar landmarks, the FAM, could be homologous to the mammalian SK. As in the BE and SK, the NAV and FAM are used independently. A PMT interpretation would also predict that the NAV and FAM can indeed be consolidated into a single integrated map. This would be important, as this issue is not yet resolved based on available data [16, 46]. Reinterpreting avian results in light of PMT would therefore be useful if the result is new predictions and new insights into this as yet unanswered question. It would also bridge the gap between mammals and birds as well as between navigation in the field and navigation in the lab. In the following discussion, the behavioral phenotype of a navigator will be described using a “+” for normal function and a “-” for impaired function. A control animal with intact function is thus BE+ SK+, whereas an animal with the loss of the BE or SK could be BE-SK+, BE+SK- or BE-SK- (see Figure four in [52] for the behavioral phenotype). Impairments can arise either from a lack of information (loss of directional cues, BE-; loss of positional cues, SK-) or from physiological impairments, for example, a bird with a hippocampal lesion, HP-, versus HP+, a bird with an intact HP.

For example, an adult HP- pigeon, released in a novel location without visual access to familiar landmarks, shows an intact initial orientation (NAV+) but is impaired orienting to the area around its home loft (FAM-). In PMT terms, this would be described as a BE+SK- phenotype. In a rat, this would be the equivalent of a CA1 subfield impairment. A BE+SK- rat can maintain a bearing, keeping its ‘sense of direction, ’ but it cannot flexibly recode a novel path using positional cues to return to a learned goal location; it has lost its ‘sense of place’ (reviewed in [52]).

Why would an apparent full hippocampal lesion spare the pigeon NAV however? One answer is that, as in the rodent, a partial function may be a sign of a partial lesion. The NAV+ ability of the HP- pigeon could be partially ascribed to this; perhaps, as in mammals, a key neural substrate of NAV lies outside of the hippocampal regions generally lesioned, such as the septum. Even within the hippocampus, Atoji and Wild [63] describe the ventral hippocampus as situated below the areas commonly lesioned (for example, studies cited in [43]). Yet it is also clear, from early gene activation studies, that it is the dorsal hippocampus, the area always lesioned, that is highly activated in adults after navigation from an unfamiliar site [66]. As currently measured, however, this activation is the summary of the entire homing event, which includes orientation from the unfamiliar site, the learning of new spatial information en route, and also the recognition and recall of already learned positional cues, as the pigeon approaches the area encoded in its FAM. How each hippocampal area is activated by these different stages of navigation is thus unknown but could be addressed with future studies using early immediate genes that examine activation both within and outside the hippocampus, after different conditions of spatial orientation. At the present time, there are too many unknown variables to be able to know what spatial functions reside outside of the standard HP lesion areas in the pigeon.

There is another explanation that is also consistent with this pattern of results, however, that does not require an exact mapping of behavior to hippocampal substructure. In mammals, the HP is necessary to create maps of positional cues or to use them in a flexible manner, to complete recalled patterns of landmarks, even if some are obscured at times during navigation. This process, the integration of the map in PMT terms, is termed pattern completion by others and is dependent on the CA3 subfield of the hippocampus [67]. But the key to this function is its flexibility. A HP- rat can use a response strategy to orient to cues that it learned before its lesion but it cannot code a new route using these cues [15]. The loss of place learning and the preservation of response learning in HP- animals has also been demonstrated in reptiles and fish [68] and in pigeons [39, 43]. The role of the hippocampus thus appears to be the encoding of spatial relations using short-term (or working) memory but it is not necessary for the recall of long-term (or reference) memory, a function subserved by other brain areas after a period of consolidation [69]. What the vertebrate navigator requires its hippocampus for is thus the flexible encoding of new spatial relations or the flexible re-coding of recalled spatial relations, as would be necessary to shortcut in a new way among familiar landmarks.

This suggests that the BE+ function in the HP- pigeon released at an unfamiliar site is due to the use of reference memory. It has been concluded that the neural substrate of the NAV must lie outside the hippocampus in the HP- pigeon [43]. What we suggest is that the NAV/BE is initially encoded by the HP earlier in development but that the information recalled at the unfamiliar site is reference memory and hence now independent of the hippocampus. To orient from an unfamiliar site, a pigeon must be able to recall the consolidated integrated map and to recognize familiar sensory inputs, such as an atmospheric chemical. Because this odor has already been associated with a direction, the pigeon can reactivate information from the BE and derive a response strategy, such as its initial bearing toward home. Upon reaching the FAM, however, the reference memory copy of the SK cannot be used in a either a simple or a flexible manner, and the bird would show a navigational impairment.

Homing pigeons develop their NAV, learning the associations of odors and winds, during their first three months of life, flying around the familiar area [26]. In PMT terms, after three months, homing pigeons should have created the integrated map and consolidated it into reference memory. If however this reference memory map is wrong, or it was never created in the first place, then a HP- bird should show a BE-SK- phenotype, that is, show a complete impairment, both at initial release from an unfamiliar site and at the home loft.

Two studies by Bingman and colleagues support this prediction. In the first [70], adult, experienced pigeons were transported 800 km from Maryland to Ohio. They then underwent HP- surgery, before they had learned any cues in the new location. They were then released at an unfamiliar site in Ohio and were highly disoriented, in contrast to the usual unimpaired initial orientation at an unfamiliar site in adult HP- pigeons. Importantly, however, the distance between the site of their initial integrated map consolidation, College Park, Maryland, and their test site, Bowling Green, Ohio, was more than twice that typically used for home navigation (approximately 800 km). Moreover, the two sites were separated by a major mountain range, the Appalachian Mountains. We submit the possibility that in this case the unfamiliar site was truly unfamiliar, in that the two sites shared no odors that were associated with a known direction. In this case, the integrated map might have been recalled but it was the wrong map. This hypothesis could be tested by varying the distance to the unfamiliar site in adult HP- pigeons.

In a second study, young pigeons were given hippocampal lesions before they had had the opportunity to fly outside the loft [71]. In this case, the integrated map might never have been consolidated. Again in contrast to HP- adult pigeons released at unfamiliar sites, these HP- pigeons were unable show an initial homeward bearing at an unfamiliar site, a result interpreted as NAV-. In PMT terms, it was a BE-SK- phenotype; the bird had not yet created an integrated map before the lesion and therefore could not recall it upon release.

One implication of the PMT framework is ontogenetic, as in the lab rat the BE appears earlier than the SK [52]. There are also sex differences in map deployment, with inputs from the SK weighted more heavily than those from the BE by female mammals, while the reverse is true for male mammals, leading to sex-specific learning strategies [72]. It is possible that a source of individual differences among pigeons in the use of directional cues such as the sun compass [16] could be sex differences in the relative weighting of directional and positional cues. For example, a GPS study of urban pigeons in Basel showed that female pigeons navigate over longer distances than males, possibly because they face higher energetic costs during reproduction. Female pigeons also forage from less variable food resources than do males [73]. Because hippocampal sex differences appear driven not by absolute home range size but by the need to use novel routes to find changing resources [74], it may be that male pigeons rely more heavily on the NAV and females more heavily on the FAM, as in some mammal species [72].

A second implication is the special role of olfaction in PMT [75]. The work of Wallraff, using simulation and measurements of odor distributions in the atmosphere, led to an important model of how pigeons could navigate using atmospheric chemical gradients, based on the distribution of ratios of odors [27, 46].

The use of odors in this way has important cognitive implications. Jacobs [75] has interpreted the similar cognitive mechanisms by which animals (both vertebrate and invertebrate) perceive odor mixtures as evidence that their brains could be creating a flexible, parallel-map-like representation. A distribution of sparse odor gradients could therefore underlie a functional topography of space, a BE constructed primarily from odors. Although pigeons can orient using only a response strategy to odors associated with direction [39, 43], Wallraff mentions that this does not rule out the possibility that a highly experienced bird could have both a topographic map and a gradient map, or a hybrid of the two ([27] page 146). Such a hybrid model could also be described as a PMT architecture [75].

The spatial interpretation of odor perception is supported by evidence from the literature that the size of the olfactory bulb co-varies with a species’ need for flexible spatial mapping, as does the hippocampus [75]. Patterns of plasticity also appear to co-vary with spatial navigation. Adult neurogenesis in the brain in mammals is primarily restricted to two locations: the hippocampal dentate gyrus, the proposed substrate of the BE, and the olfactory bulb. The size of both the hippocampus and the olfactory bulb in vertebrates may co-vary with a species’ reliance on odors for spatial navigation [75, 76]. Thus olfaction maintains not only an ancestral position in the evolution of spatial navigation but possibly also a primary position, not just in pigeons but in all animals. Further, the importance of olfaction in pigeon navigation is concordant with all of the analyses and predictions of the original and expanded PMT [56, 75].

In conclusion, there are several advantages to considering a PMT framework for pigeon navigation. First, it offers a more specific model to integrate concepts from two dominant research paradigms, homing pigeons in the field and rodents in the lab. The PMT framework integrates the question of navigation at multiple levels of analysis, from its development (ontogeny, sex differences) to its mechanism (BE, SK circuitry) and phylogeny (ancestral BE, derived SK). The great strengths offered by studies of pigeon navigation could therefore have profound implications for understanding the BE and SK of the rat in a laboratory maze. At the same time, a PMT framework can propose new ways in which the pigeon NAV and FAM might be integrated and how this could be tested.

In light of the striking similarities between the olfactory systems of vertebrates and invertebrates, in particular the well-studied flying insects [77], PMT also suggests a framework to be considered in the study of invertebrate navigation. The study of navigation in invertebrates is particularly interesting in this context as the species under study have so far either been studied in their natural habitat or in semi-natural laboratory conditions, under highly reduced stimulus conditions. And since nothing is known about the neural structures and processes underlying navigation in these species, it is not surprising that there is an ongoing debate about the role of elemental versus highly integrated forms of navigation among invertebrates [20]. Because we expect that no solution will be reached until the neural mechanisms are uncovered, given the recent demonstration of map-like behaviors in honeybees [33], it is possible that insect navigation could also be profitably analyzed in a PMT framework, with BE and SK functions. For example, the time-compensated sun compass appears to be housed in the central complex of insects, at least in the locust [78]. Since a subtype of neurons in the central complex compensates for the diurnal shift of sky polarization pattern, this structure needs to integrate information from the circadian clock and visual information, allowing it to calibrate the sun compass. Thus it is likely that visual gradients in addition to celestial cues are either analyzed by the central complex or are retrieved from other parts of the visual system in the insect brain, such as the optical tubercle. Such an integration of directional cues suggests a BE function. A different structure of the insect brain, the mushroom bodies, are known to process highly integrated sensory input and to contribute to cross-modality memory processing [79, 80]. Object recognition would be a component of the insect SK, and it is likely that this is processed in the mushroom bodies [81]. In essence, the insect brain may code spatial components separately for BE-type functions in the central complex (e.g., sun compass, gradients) and SK-type functions in the mushroom body. An additional analogy to the conditions in the vertebrate brain can be seen in the fact that the central complex comprises a rather stable and evolutionarily ancient component of the arthropod brain, whereas the mushroom body is highly variable between different arthropod lines and adapts flexibly to ecological constraints.

### What the lab can learn from the field

If the lab-derived PMT framework can offer new insight into patterns of navigation in the field, then what can this integration of lab and field do for the design and interpretation of studies in the lab? (Note: we will use PMT terminology from this point forward; the semantics of cognitive mapping and navigation is treacherous territory [27], see discussion in [82, 83]). First, how can this framework be used to understand the controversy regarding whether or not a lab rat can shortcut, in other words use an integrated map, in a lab maze? To behave flexibly in this way requires, as already discussed, not just an intact HP, but an intact BE. For an animal to express and use the BE, it requires a sensory environment with reliable directional cues [52]. The directional cues used by honey bees and homing pigeons are quantitatively and qualitatively different from those available to the laboratory rodent. It is this disparity, we will argue below, that leads to failures to show flexible shortcutting in the lab. We will first review this literature and then discuss the implications for our understanding of spatial behaviors in the lab.

#### The difficulty of demonstrating map-like behaviors in the laboratory

The combination of new map concepts, new tools for understanding behavior under natural conditions and new tools for understanding the brain in the lab has led to remarkable successes. What remains a puzzle is that behavior-only demonstrations of a ‘cognitive map’ (or integrated map, as we define it) in the laboratory rat [13, 84] and in humans [85], the two species in the title of Tolman’s original paper [2], remain controversial and elusive.

Part of the confusion may arise from different uses of the term ‘cognitive map, ’ even among psychologists studying rats. There are several forms of problem in the lab. For example, a rat moving in an open space is able to shortcut if it is displaced to a new location or if a familiar route is blocked. Such results using a water maze, a round pool of opaque water that hides a submerged goal platform, were interpreted as support for the cognitive map concept [51]. Rats were first trained to swim along a fixed path to a visible platform in the water maze. Later, the platform became invisible and finally the rat was released, in probe trials, from new points around the 200-cm-diameter circular maze. The rat had continual access to extramaze cues during all phases of the experiment. The rats’ ability to take a direct path—in other words, a novel path—from the novel release point was demonstrated in HP+ rats, but HP- rats were unable to do this, although they could learn the fixed route during training. These results demonstrated that a rat can take a novel route after displacement.

Benhamou, however, pointed out that the HP+ rat had a full complement of familiar landmarks during the test and therefore could simply have been ‘piloting, ’ which he defined as orientation to known cues [84]. Piloting is defined more precisely in pigeon navigation as “guidance by familiar landmarks” ([27] page 4), though their actual route may be influenced both by information from piloting and from compass information [16]. In PMT terms, piloting is also navigation using positional cues within a SK, independent of directional cues. However if an animal was navigating within a consolidated integrated map, then moving among familiar positional landmarks (i.e., SK integrated to BE) could involve both types of cues. In other words, one can navigate to positional cues within a SK or within an integrated map, and the use of directional cues will be absent in the former and present in the latter.

**Figure 3:**
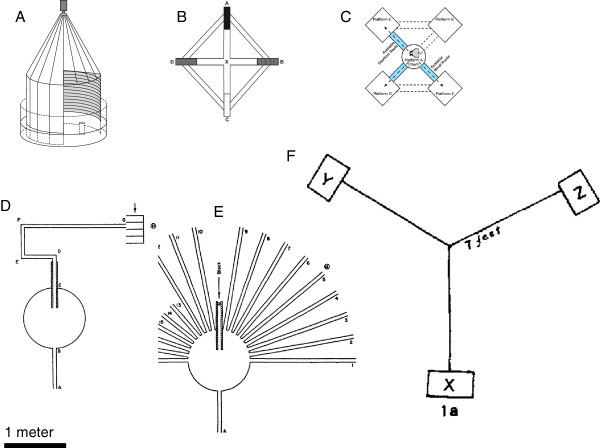
**A comparison of mazes used to study map-like behavior in the laboratory rat, drawn to the same scale. A**. Marquee water maze of Benhamou [84]. **B**. Enclosed tunnel kite maze of Roberts et al. [89]. **C**. Enclosed linked box and alley maze of Grieves et al. [13]. **D**. Training maze of Tolman et al. [94]. **E**. Test maze of Tolman et al. [94]. **F**. Three-table maze of Maier, 1932 [91].

In both cases, positional cues are needed. Benhamou eliminated piloting, by his definition, in a water maze experiment. HP+ rats oriented to a hidden platform in a 120-cm-diameter water maze where the pool was surrounded by a marquee that could be rotated to partially block the view of extramaze landmarks during the test trial (Figure 3A) [84]. With access to only a novel subset of extramaze cues for the test, rats could not orient significantly above chance to the hidden platform [84]. This result demonstrated that these rats had been piloting via positional cues only and not using an integrated map, which would have allowed them to compensate for the missing cues.

As Benhamou points out, however, piloting cannot explain results obtained from golden hamsters, returning after exploration to a nest attached to a large arena [86]. HP+ hamsters first lived in a 180-cm-diameter round open arena with limited extramaze cues. They were then motivated to collect food from one of four identical feeders and take a straight path back to their nest to hoard the food item. They were lured to different locations in the arena, by following a lure presented by the experimenter, before their journey to the reward location and subsequently to the nest location. Under these conditions, hamsters showed extremely precise orientation, by the presumed mechanism of vector addition [86]. In theory, vector addition could be used at any scale as a mechanism underlying integrated mapping. This study demonstrates that a lab rodent can use vector addition to shortcut, in the absence of pilotage. In PMT terms, the hamsters were living in the apparatus and presumably possessed a detailed, consolidated integrated map (BE+SK+), with consolidated directional and positional cues, enabling the hamster to use its hippocampus to make flexible calibrations. Vector addition could then have been the mechanism they used to extract the relevant information from their integrated map.

Despite this demonstration in hamsters, concerted efforts to show shortcutting in the lab rat have only succeeded under certain conditions. To control the paths travelled, most of these experiments have used mazes constructed from intersecting, closed tunnels, to exclude extra-maze cues, rather than open fields, as in the water maze and hamster experiments above. Although it is true that rats under natural conditions orient in closed burrows, they also forage over hundreds of meters in open air, with full access to odors and wind [87]. In other terrestrial mammals, such as carnivores, the relative size of the olfactory bulb co-varies with home range size [88], which suggests that non-avian vertebrates also rely on olfaction for navigation [75]. If rat navigation is similar to pigeon navigation in this way, then access to such cues may make a critical difference in the rat’s ability to use its integrated map. It is possible and even likely that a rat uses different mechanisms in the overlearned, 3D underground maze of its burrow compared to navigating across novel 2D surfaces in foraging and mating expeditions.

Nonetheless, even in mazes constructed from tunnels, rats can take shortcuts if a known route is blocked. But in most cases, this only occurs if the rat has already traversed the shortcut route in its exploration of the space before facing the now-blocked familiar route. One example of this is a kite-shaped maze (approximately 160 cm in diameter; Figure 2B). This was used to test integrated mapping without pilotage by excluding visual access to extramaze cues [89]. Across experiments, rats only chose the correct arm for a shortcut if they had previously traversed that arm in the past.

A similar result was obtained using an alley maze when extramaze cues were excluded (Figure 3C). Grieves et al. [13] adapted their maze from a study on spatial insight in human children, in which children were asked to shortcut between previously disconnected rooms [90]. In the rat version of this, once again rats only chose the correct shortcut if they had previously walked down that arm during training. As the authors discuss, this result is surprising for two reasons: first, because the problem appeared to be similar to that solved so quickly and accurately by golden hamsters [86]. Second, because the neurophysiological evidence from the lab rat suggests that they have all the information required to make this vector addition. The authors suggest that the result could be a consequence of the difference in familiarity of the route: in an open arena, such as that used for the golden hamsters, the animal had previously traversed all possible routes in its normal exploratory behaviors. In the alley and tunnel mazes for rats, in contrast, such exploration is limited by the experimenter [13].

The need for a rat to experience all the possible routes in a space before shortcutting within it was also demonstrated in an open surface maze, the Maier three-table task [91] (Figure 3F), three tables linked with bridges. It was an early demonstration of latent learning, which is exploratory behavior in a space with no extrinsic reward for such exploration [2]. In the Maier task, rats are fed on one table and are then tested by being placed on another table, to see if they are able to choose the correct bridge to return to the feeding table. The apparatus is large (approximately 215 cm in diameter), the surface is open to the air and all extramaze cues are available. Paul Ellen and colleagues later demonstrated that lab rats could only solve this task under certain conditions. If allowed access to only one table and its associated bridge per day, or access to just the tables or just the bridges, rats could not choose the most direct route on the test day. In contrast, rats given access to at least one pair of tables and their associated bridges per day for three days were able to choose the correct route above chance on the fourth day [92, 93]. Thus despite a rat having access to all extramaze cues, and hence the ability to pilot, rats were not able to integrate their experiences when they had access to only one piece of the apparatus per day. This can be re-stated in the terms of PMT: each table was an isolated SK but they were not integrated and recoded in BE coordinates. To do this, the rat needed to integrate BE and SK into a working memory representation of the integrated map, which it could only do if it was allowed to traverse all three bridges within a single exploratory bout.

Even so, the requirement to traverse the path before it can be used later as a shortcut may not be a universal requirement for the lab rat. Again, it may be the details of the experiment that determine whether the rat succeeds or fails. In 1946, Tolman and colleagues published their sunburst maze experiment [94] (Figure 3D). Lab rats were trained to follow a meandering alley that led across a circular platform, through a visually shaded alley and then to an open alley, which led to a food source; the apparatus was approximately 4.5 m in length (Figure 3D). The only source of light in the room was a 5-watt incandescent bulb in a desk lamp, which was tilted to shine down the final alley approach to the reward. After 5 training trials, the apparatus was rebuilt into a configuration that blocked the original path and instead opened up a fan-like array of alternatives. Olfactory cues were controlled by rotating the circular start board and the dim light was repositioned such that it now was closer in proximity to an adjacent alley, not the alley that led to the location of the food. Rats were given one test trial; the majority of rats showed evidence for an integrated map strategy: they chose the alley that would have led to the food during the training trials, not the alley that led to the light, which would have been a simple response strategy.

This experiment is often criticized for an apparently obvious methodological flaw: the light served as a beacon to guide the rat’s orientation to the correct alley [14, 83]. Yet the authors address the point, pointing out that the light had been moved to rule out a simple beacon approach to the light source. The sunburst design was also replicated in two species of wild rodents (voles), where it was used to study the species and sex differences predicted a priori by the species’ spatial ecology [95], a result subsequently replicated using other vole species navigating symmetrical mazes [96]. Despite this, the sunburst maze experiment is usually discredited [83] or ignored in discussions of navigation. Yet it appears to be a robust demonstration of a lab rat taking a novel route to a known location and, because of the low light in the room, probably doing so using few distinct visual landmarks. Notably, the rats were trained in fewer than 10 trials; this is rapid, and possibly more natural, spatial learning compared to rats learning in smaller, semi- or completely enclosed mazes where tens to hundreds of training trials are required [13].

Another possible reason for the discrepancy in findings across studies could be the physical scale of the experimental apparatus in Tolman’s study compared to those used in more recent work. As we will discuss below, the absolute scale of a space affects the information that can be extracted by a navigator. Figure 3 suggests there could be a historical trend in which tabletop mazes used to study navigation in rats have become smaller and more closed to the air. Rapid spatial learning, as in Tolman’s sunburst maze, Maier’s 3-table maze and Etienne’s hamster arena, might only be found in spaces that are not only open to the air (and hence to volatile odors) but also where the animal is moving in a space much larger than its body size [86, 94, 97] (Figure 3A-C). Both of these variables—restricted access to volatile odors and smaller arena size—would create a BE-SK+ task environment, even for a HP+ rodent [52]. In the absence of the BE, it would be difficult to create shortcuts. However, even in a small, enclosed maze, the rat could encode a BE if it had the opportunity to traverse all paths and therefore map directions using path integration.

One could argue that such a solution is not integrated mapping—if this is defined as a navigator creating a completely novel path. Yet no agent can navigate in the absence of all familiar cues; it must have some association between a goal and a cue. Perhaps what is needed is not more discussion of experimental details but an emulation of pigeon navigational methods by rat researchers. For example, one could develop methods inspired by pigeon GPS studies, in which a rat could be tracked exploring one part of a large exterior space and then be released at an unfamiliar location, where familiar visual cues were not accessible (SK-) but familiar odor gradients were (BE+). The same manipulations of HP lesions and olfactory system impairments should then result in replication of the same pattern of olfactory-dependent integrated mapping as that seen in the pigeon orienting over natural landscapes. Jacque Bovet’s experiments with red squirrels using a similar paradigm suggest that like pigeons, squirrels show a BE+ phenotype, orienting initially in a homeward direction [28].

#### The importance of self-movement for the integration of spatial cues

Even in a small lab space, there is evidence that the scale of the space has implications for the mechanisms underlying the use of a spatial strategy, in particular the integration of directional and positional cues as in an integrated map. A canonical example is the effect of scale on an animal’s ability to integrate a directional cue, a polarized arena geometry such as a rectangle, with a positional cue associated with a reward. Ken Cheng demonstrated that a rat who had previously learned to find food in one of four corners of a rectangular arena would, upon vestibular disorientation, ignore the positional cues found in the corners and orient instead only to the two corners that were geometrically equivalent. One interpretation of this result is that the rat possesses a geometric module that can be disassociated from other cognitive mechanisms, although there are also other interpretations [98].

Nevertheless, the study of the integration of a space’s geometry and the features found within the space (such as a colored wall) has been the focus of intense comparative work, with studies of fish, birds (both chicks and pigeons), laboratory mammals, and human children and adults. One generalization that has emerged from this work is the effect of scale on this integration; the integration process differs between a large and a small version of the same experimental arena. The general pattern that emerges across species is that animals are more likely to encode a location using the geometrical properties of a larger space than those of a smaller space [99].

One reason for this effect might be the effect of scale on measuring distance using self-movement. A polarized space, such as a rectangle, offers a directional cue whereas a symmetrical space, such as a square, does not confer directional information. The disassociation of geometry and feature in these experiments thus could be an example of the disassociation of a directional cue from a positional cue, that is, the BE from the SK [52]. If so, then scale suddenly matters very much, as a navigator’s accuracy in measuring the relative length of the two axes will be determined by the absolute size of the space in relation to its body size. It should be harder to measure the differential length of walls in a smaller than in a larger room, because the navigator has less sensory information (e.g., optic flow) and fewer samples from which to measure the distance.

The effect of scale on the ability to integrate geometry and feature has been found in redtail splitfin fish and human children, who may integrate the cues of geometry and feature in large spaces but are less likely to do so in small spaces [100, 101]. Two bird species differ from this pattern, as domestic chicks and laboratory pigeons are able to use both geometry and feature in all arenas, regardless of size. However, in chicks the degree of reliance on each cue type can be quantitatively disassociated in large and small arenas: if features are removed, chicks will search in geometrically correct corners. But when faced with more extreme geometric distortions, the chicks, like children and fish, show more geometric errors in the smaller space [102]. When one or the other cue is removed, quantitative differences emerge, with chicks appearing to rely more heavily on geometric information in smaller than in larger arenas [103, 104].

There are clear species differences, with adult fish relying more heavily on geometry regardless of context than chicks, who always attend to feature. Such differences could be the result not only of phylogeny but of ontogeny, as it is a comparison of an adult fish with a juvenile bird. A fish also maps space in 3D while a chick, too young to fly, must map space in 2D [99]. This dimensional change from 2D to 3D mapping could well affect hippocampal encoding [105, 106].

The predilection of the chick for features is also interesting in light of the general rule that directional cues are weighted more heavily than positional cues [99]. One explanation for the discrepancy in the chick is that the domestic chick, as a precocial juvenile that feeds itself by imprinting on and following the adult hen, may not need to map allocentric space, instead relying on a response strategy (‘follow hen’) for foraging decisions. However, the chick must be able to identify food and predators, both of which could be encoded as highly salient positional cues. This functional explanation for the chick’s bias for encoding feature over geometry (i.e., positional cues over directional cues) could be tested via longitudinal studies of precocial bird species, to determine whether a flying adult relies more heavily on directional than positional cues relative to a walking chick, and if so, whether this difference emerges with independent flight. This would also be a reversal of the pattern seen in the lab rat, in which the BE emerges earlier in ontogeny, before the SK and the integrated map [52].

In summary, we have suggested that the absolute scale of an experimental space may directly affect the precision with which an animal can estimate directional cues. With a less reliable BE, the animal should preferentially encode locations on a SK. Manipulations such as vestibular disorientation ‘break’ the integrated map by eliminating the internal directional cue needed to calibrate the SK with the BE. With no integrated map, the meaning of a positional cue is lost; the animal is reduced to a single directional cue, the geometry, which creates a BE+SK- phenotype and the animal, using geometry alone, cannot distinguish between geometrically identical corners. This scenario is greatly simplified in light of the actual data, in which results can vary by species and age [99]. But this suggests that because of the primacy of the BE in integrated mapping, it is not only directional cues but the scale of directional cues that may be critical for their accurate encoding. We now discuss the significance of this point and its corollaries, including the effects of locomotion, experience and motivation, on integrated mapping in the lab.

### Why the lab needs to consider natural movement

To solve the integrated map problem, an animal must have access to stimuli associated with its goal location. Because diverse species, including honeybees, homing pigeons and fruit bats, can orient in the absence of learned positional cues, the integrated map solution must be based on previously learned directional cues. These cues must not only impute direction, as would a sun compass, but must also impute the identity of the specific direction of the goal, usually home. This could be achieved in simpler or more complex ways. In a simpler method the navigator could detect its absolute location on an extended cue such as a gradient of a known odor or an extended landmark, such as the edge of a forest or a road. A more complex solution is the calibration of these directional cues in relation to each other in a BE [52], where odors could theoretically supply a rough topography [75]. In both cases, to solve the integrated map, the navigator must be able to reconstruct its location in relation to familiar directional cues, including extended cues. Unlike the encoding of discrete objects, an extended cue must be modeled and encoded as the spatial derivative of repeated sampling at known temporal intervals. To do this, the navigator must be able to systematically sample the spatial distribution of the magnitude (e.g., chemical concentration, size, length, or volume of a sound source) of the directional cue, using an internal pacemaker and self-movement [52]. It is by repeated sampling in a known spatial pattern, such as the characteristic circular orientation flight seen after release in displaced flying animals, that the animal can define its current position in relation to learned directional cues.

At the same time, it is clear that pigeons can recognize an odor and recall the direction associated with that odor without any self-locomotion at the release site. All that is needed is that the pigeon has access to an odor that it has associated with a wind direction for accurate orientation [27]. Such orientation would clearly be a response strategy. Still, using a response strategy under certain experimental circumstances does not mean that pigeons cannot also use more complex and flexible strategies, such as a combination of response and place strategies, under other experimental circumstances, as do rats in lab mazes.

To solve the integrated map problem, the navigator must sample at least two, or perhaps several, directional cues in sufficient detail to determine its current location. The space covered to complete these samples will be determined by the cues: the more quickly the magnitude of a cue changes across space, the less space the navigator must traverse to determine its direction. If a directional cue changes very slowly across space, however, the navigator will need to traverse a greater distance to collect and integrate sufficient samples to estimate the polarity of the cue. Without the opportunity to collect the data to map self-movement in relation to the directional cue, the animal cannot solve the integrated map problem.

Thus the absolute amount of self-movement could critically constrain the accuracy with which the directional cue can be measured and hence the spatial resolution of the animal’s integrated map. Therefore self-movement may be critical for tasks in which cues must be integrated. This could include overt mapping tasks, such as the 3-table task or the sunburst maze, but could also include simpler versions of integration of directional and positional cues, such as integrating the geometry of a space with its features. If directional cues are absent or unreliable, or the animal has insufficient experience using these cues to encode direction and distance, then it may be difficult for it to use an integrated map strategy. This might be why the integrated map test fails under some experimental conditions, as we discuss below.

#### Scale and physical structure

A male lab rat in standard housing conditions lives in a 0.15 m^2^ world, under unchanging conditions of light, temperature and humidity. In the field, male Norway rats have been released individually on a rat-free island, where they travelled an average of 86 m per hour while exploring and, after 3 weeks, were exploring 90% of the island’s 9 ha surface per night [87]. Not only is the scale of movement in the field larger, but movement is carried out in the context of a rich complement of directional cues (Figure 1). These could include extended cues such as gradients, constructed from changes in stimulus intensity experienced with self-movement (e.g., odors, magnetic fields). An extended cue could also be a linear landmark, such as an extended object with a longitudinal axis (e.g., a river, a line of permanent vegetation). The physical slope of the terrain can also be used as a directional cue [107, 108]. Lab pigeons may even use slope as a bicoordinate representation in terms of the slope’s vertical (up-down) and orthogonal (left-right) dimensions [109].

In the lab, the environment is smaller, simpler, more symmetrical and less diverse than a field ‘navigational Umwelt’. Lab environments are also designed to be coded in terms of visually distinctive cues, even for species such as lab rats which have keen auditory and olfactory acuity. Hence, by design, the laboratory environment contains significantly less information for integrated mapping and may present a more difficult task to a navigator than does the larger and richer sensory world of the field.

#### Locomotion and sensory input

If an animal is moving over larger distances it can extract more information from the environment. Honeybees can use an extended landmark, such as the edge of a forest, as a directional cue and in fact can use this physical structure to replace their use of the default directional cue, the sun compass. The larger scale and the availability of extended cues in the field thus increases the distance moved and information gained. The increased scale of the field is reflected in the multiple processes used for measuring distance in the field. For example, in reduced environments that mimic lab conditions, the honeybee measures distance by a stereotypical odometer based on optic flow, but in natural conditions the sequence of landmarks is also used for range estimation [110].

Because of the effects of scale on measurement of extended cues, the mode of locomotion could affect the use of stimuli in navigation. In the field, even in fairly barren landscapes, there are many directional cues, from the reliable movement of celestial objects through the sky to the gradients of geomagnetic fields of the earth. Because voluntary movement, by which distance can be encoded, is necessary to extract metric information from such sources, an animal’s mode of locomotion must affect the cues that it can use for this. A pigeon, with high visual acuity, flying not only high but swiftly above a landscape, can extract more precise information about distances than a walking, terrestrial rodent, because the pigeon can sample over a larger range of values of the stimuli. A flying insect, such as the honeybee, similarly collects more information than a walking ant; and while it might fly at a lower elevation than a bird, a honeybee may still cover the same or even greater absolute area. Thus aerial animals (pigeons, honeybees) may sample and map extended cues in qualitatively different ways than their walking counterparts (rats, ants). An aquatic animal, such as a fish, may face a situation similar to that of an aerial animal, in which it can observe the landscape from different elevations. Fish may rely more heavily on positional cues where it swims more slowly, in a benthic, or bottom, environment but more heavily on directional cues where it swims more quickly and farther in a pelagic, or open water, environment. Again, the mode of locomotion and the speed of movement should have implications for the mapping abilities of closely-related species that live in different aquatic or aerial habitats. Species that move quickly over the same landscape might be more likely to use directional cues and hence a BE and integrated map strategy than species that move less quickly and over smaller distances.

By this logic, the absolute distances covered by an animal could have a direct effect on its ability to extract information from its environment. By multiplying distance traveled by the animal’s lifetime navigational trips, it should be possible to estimate the amount of information gained during navigation in an animal’s lifetime. It has been estimated, for example, that an individual of the desert ant species, *Cataglylphis fortis*, may make no more than 30 foraging excursions in its entire life [111]. It may be therefore that the vertebrate and invertebrate species that have been considered model organisms for the study of spatial navigation, the walking Norway rat, house mouse and desert ant, may in fact be the species least likely to use an integrated mapping solution to solve a navigational problem.

#### Motivation and strategy cost

As Samuel Johnson was quoted, “Depend upon it, sir, when a man knows he is to be hanged in a fortnight, it concentrates his mind wonderfully” [112]. Under natural conditions, animals explore continually to track the distribution of resources for survival (food, competitors, safety) and reproduction (mates, competitors). Becoming lost in this world likely leads to aggression from other residents, reduced foraging efficiency and even starvation, injury and death if the animal cannot establish a new territory or home range. None of this will (or should) occur in the lab; being disoriented in the lab may lead to a momentary frustration, shock or anxiety, but animals reared and kept under constant conditions probably learn as adults that this aversive condition will be of short duration. Even the water maze, which initially must be perceived by an animal as a life or death situation, becomes a routine task for the semi-aquatic Norway rat, who appears to learn that a certain duration of swimming will always result in rescue by the experimenter. Thus, in addition to fewer cues in the lab, there may also be lower motivation to learn to use these cues.

Knowing that an animal is orienting under conditions of high motivation is obviously critical for disentangling knowledge from performance, as in latent learning [2]. It must therefore necessarily be the case that the content of learning is more easily seen in the field, where motivation must be significantly higher than in the lab. If motivation in the lab is lower, then an animal may require more training to learn a specific navigational strategy. Specialized and intensive training may inadvertently lead an animal to different forms of spatial memory and different heuristics for performance than that intended by the experimental design. Rapid spatial learning may indeed be the rule under natural conditions, as it is rapid in wild desert ants [111] and fox squirrels [113].

Another motivational issue is related to the costs and benefits of a strategy choice in a particular apparatus. A lab rat may be less likely to take a novel tunnel that is the ‘correct’ choice for a integrated mapping strategy because of the potential costs of entering an unexplored space, which could house new and unknown dangers. Even if the rat has access to mental models of multiple routes, it may choose to take a longer but already reconnoitered and hence less risky path.

This issue of strategy choice in light of potential risk has recently appeared in cross-species comparisons on a task measuring physical insight, the trap tube or trap table task. This task is designed to measure an individual’s ability to reason about physical causes, as inserting the tool at the wrong end of a tube causes the reward to fall into a trap and become inaccessible. The failure of many species, including tool-using capuchin monkeys, on this task appeared to be evidence that such physical insight was lacking in these primate species [114]. In response, Silva and Silva tested adult humans on a similar task and found the same pattern of performance as in the monkeys, despite the subjects’ obvious understanding of the physics of the apparatus. Humans adopted a highly risk-averse strategy, appearing for all intents and purposes as if they did not understand the physics of a simple mechanical problem [115]. Using an integrated map strategy may be a similar example of insight into a physical problem that can be solved in multiple ways. Without studies that vary the costs and benefits of different strategies, both energetic and emotional, we cannot assume the full range of an animal’s behavioral repertoire.

Another cost is that of developing and maintaining the physiological substrates of learning. If an animal has a less well developed or flexible neural architecture for a cognitive strategy then this must increase the relative cost of that strategy. Because lab animals have reduced sensory inputs and motor outputs, as well as lower motivation, they have less complexly structured brains [116]. The importance of environmental enrichment for spatial learning has been well documented in lab rodents (even if ‘enrichment’ means less radically impoverished than standard housing conditions). Similar effects of experience on neural development are seen in the honeybee [117, 118].

Compounding this effect, another physiological parameter that is known to handicap the cognitive performance of lab animals is the mismatch between cognitive load and active cycle. The ancestral species of the lab rat, mouse and hamster were nocturnal, yet many studies are conducted during the diurnal period of inactivity, despite evidence for time-of-day effects on memory [119]. Therefore, not only is the brain of a laboratory rodent likely to be compromised by an artificially impoverished environment, both in terms of physical and social structures, but its performance may be further handicapped by the physiological consequences of the lab testing schedule. There have been no direct comparisons of spatial cognition between lab rats and lab mice and their conspecifics raised and tested under natural conditions; we suggest that such a study might reveal qualitative differences in the use of spatial strategies such as the integrated map.

#### The impact of movement on calibration

Finally, it is the nature of spatial representation to combine multiple, redundant sources of information. Because of the greater richness of independent cues in the field, spatial representations in the field probably can be encoded much more redundantly than in the lab. This redundancy should make field representations more flexible and hence more powerful, as the navigator can more easily calibrate its position in the face of distortions and hence discrepancies among different information sources.

Animals navigating in the field not only have more cues with which to perform this calibration but also have more experience tracking changes, as many of the directional cues in the field are also highly dynamic. Celestial objects are mostly in constant motion. Odor gradients and even magnetic fields are subject to daily and seasonal fluctuations [120, 121]. Such dynamism demands calibration to maintain the integration of directional and positional cues, maintaining the conditions necessary to use an integrated map. In the case of magnetic fields and other extended cues, there may also be daily and seasonal fluctuation in the level of noise in the signal, which must be learned and taken into account. These demands may explain why only experienced, adult migratory birds, but not naive juveniles, can compensate for experimental displacement during navigation [41]. Juveniles lack the experience to have encoded the multisensory information necessary to construct an integrated map. The learning involved may at times have the character of imprinting, allowing non-migrating animals to learn only one sun azimuth/time function. For example, a honeybee cannot adapt its sun compass to a location north of the equator if it had calibrated its sun compass at a location south of the equator [122].

Navigation in the field is thus characterized by the redundant encoding of both directional and positional cues. Directional cues are often weighted more heavily than positional cues, even when doing so means tracking dynamic cues, as in the sun compass. The ability to draw on such disparate classes of orienting stimuli allows a navigator to represent different scales of the environment at high resolution. For example, a forager might use its memory of locations relative to the bearing map—that is, a coarse, large-scale map—to orient to patches where food has been discovered. When making the decision to choose between patches, however, it may use multiple different classes of cues, extended cues and discrete cues. When foraging within a patch, it may switch from a BE to a single SK, orienting to small-scale local landmarks within the patch, to monitor its foraging success and make patch harvesting and departure decisions. Lab studies where the animal has no need to track dynamic stimuli, to calibrate stimuli or to explore and integrate different scales of movement might well grossly underestimate an animal’s spatial abilities.

## Conclusions

Animals must navigate efficiently and predictably in an unpredictable world. With the development of new technology, both in the field and the lab, the answers to fundamental questions about navigation may suddenly be within reach. Such research will be of conceptual importance for movement ecology, cognitive biology and neuroscience [123, 124], but will also be of pragmatic importance for artificial intelligence, robotics and conservation biology, as all of these fields require knowledge of what animals know about space and how they use this information.

In this review, we have tried to address the question of what concepts are missing in each of several research paradigms for studying navigation and how cross-pollination between these paradigms could enhance research progress. For example, we proposed that the conceptual gap between two dominant paradigms for animal navigation, the homing pigeon and the laboratory rodent, could be bridged using concepts defined in PMT, a navigation model developed from lab data. The importance of a bridge between these paradigms has been long acknowledged [125], but what has been missing is a common language and common model. If the NAV and BE, the FAM and SK, are indeed convergent, if not homologous, then behavioral concepts from the development and function of pigeon navigation could be applied directly to navigation in lab rodents. Likewise, selective BE and SK impairment methods developed for the rodent could be adapted to understand the dual mechanisms of navigation in pigeons and other free-ranging vertebrates, such as bats.

Such cross-pollination might also heal divisions within the discipline. There has been a strange schism in the field of navigation, with modern behaviorists skeptical of mapping solutions while neuroscientists readily use such concepts to drive their research. This debate, a form of which has been ongoing since Tolman’s original work on rats, evokes the eternal question of the parsimony of simpler mechanisms. But as Albert Einstein wrote, “It can scarcely be denied that the supreme goal of all theory is to make the irreducible basic elements as simple and as few as possible without having to surrender the adequate representation of a single datum of experience” [126]. We do not yet have enough information on the neural substrates to determine which is a simpler and more economic computation, indexing multiple isolated memories or creating an integrated spatial memory. The question may not be which explanation is formally more or less simple, but rather what we are searching for when probing the brain of a navigating animal. A model too simple may fall short of the rich computational power of the navigating brain, which, after all, maybe be the reason the brain evolved in the first place [75].

The behaviorist’s reluctance to embrace mapping strategies may stem not only from the constraints of the lab but also their very choice of study species. Differences in locomotion, both in terms of speed and dimensionality of space, as well as the absolute quantity of cues encoded over an animal’s lifetime, could all have significant effects on a species’ choice of navigational mechanism. Thus study species, and their consequent navigational Umwelt, could be a fundamental determinant of the outcome of navigation experiments. If an animal such as the desert ant *Cataglyphis* is studied in an environment lacking extended landmarks, and each individual performs a rather limited number of foraging/exploration runs, then it is not surprising that only rather limited navigational strategies can be detected. Focusing on invertebrates that display complex, flexible navigation in the field must be the next step toward models of navigation that apply to both invertebrates and vertebrates and that may have evolved through convergent evolution.

If a species’ sensory abilities in the lab impose artificial constraints on navigation, it is possible that the cognitive or integrated map debate may be largely an artifact of the lab. We must determine what is necessary to solve the integrated map solution and deliver these conditions to animals, wherever they are navigating. Not only must the information from these cues be available, but they must be used by an animal that can express its natural potential for information processing. This must include, first, a motivated brain, second, a brain that has developed in a suitably challenging and unpredictable environment and third, the absence of unnatural levels of overtraining. Only under these conditions would one expect the animal to have the options from which to choose, when needed, a difficult and risky strategy such as integrated mapping across a novel terrain. As in the classic demonstrations of latent learning, only then will the navigational demands be high enough to unmask ‘insight’ strategies such as shortcuts.

Robust spatial navigation is based on redundant information sources, with multiple frames of reference and sensory modes that can serve for calibration and back-up. Without the information or the information processing power to create and use an integrated map, the optimal navigator must needs choose another strategy. In short, only when animals are orienting in their natural environment or a laboratory setting close to it, with the cues necessary for navigation and the motivation necessary to use these cues, would one expect their full repertoire of spatial strategies to be revealed. A synthesis using a theory grounded in evolution, such as PMT, with its specific predictions about an animal’s use of directional and positional cues, could integrate concepts obtained from flying and walking species, lab and wild animals, invertebrates and vertebrates.

The lab and the field have much to teach each other. The field can inspire the lab to increase the complexity of the experimental environment, so that it does not distort its models based on a subset of stimuli, missing opportunities to model the true complexity of animal thinking. Field studies may also help the lab to interpret paradoxical results, such as the preplay of place fields in unexplored space, which may be a signature of an animal’s use of the BE to extrapolate vectors from extended cues. The neuroscience lab may, reciprocally, have much to teach the field, at least to direct the kinds of questions that are asked of animals under natural conditions. Field behaviorists could replicate the experiments from the lab, to determine how moving from constrained to unlimited spaces changes the hierarchy of spatial strategies that are used. The impact of locomotion mode on the nature of the spatial representation could also be studied in the field, using new technology to track and test spatial behaviors, across a much wider range of taxa than is possible in the lab, and employing methods that are well characterized from the lab. The same questions could then be brought back into the lab, where studies could use virtual reality arenas to compare disparate taxa navigating across different scales of space and using different forms of locomotion.

The last three decades have produced a seismic shift in the quantity and quality of available data and the new insights into the age-old question of the cognitive map. Now is the time for greater collaboration across the behavioral disciplines, with researchers moving towards a full integration of the concepts and methods that will lead to new understanding of how the mind maps the world.

## Authors’ information

LJ is Professor of Psychology and Neuroscience at the University of California, Berkeley, has an office in Tolman Hall and studies cognitive evolution, in particular the evolution of navigation and its neural substrates. RM is Professor of Neurobiology at the Institut für Biologie, Freie Universität Berlin. His research program is centered around the neuroscience of cognition in the honeybee, with a particular focus on memory processes and spatial navigation.

## Abbreviations

NAV: Navigational map
FAM: Familiar area map
PMT: Parallel map theory
BE: Bearing map
SK: Sketch map
HP: Hippocampus
+: Structure or function intact (e.g. HP+, intact hippocampus)
-: Structure or function impaired (e.g. FAM-, impaired familiar area map).
